# Lack of global meiotic sex chromosome inactivation, and paucity of tissue-specific gene expression on the *Drosophila X *chromosome

**DOI:** 10.1186/1741-7007-9-29

**Published:** 2011-05-04

**Authors:** Lyudmila M Mikhaylova, Dmitry I Nurminsky

**Affiliations:** 1 Department of Anatomy and Cellular Biology, Tufts University School of Medicine, Boston, USA; 2 Department of Biochemistry and Molecular Biology, University of Maryland School of Medicine, Baltimore, USA

## Abstract

**Background:**

Paucity of male-biased genes on the *Drosophila X *chromosome is a well-established phenomenon, thought to be specifically linked to the role of these genes in reproduction and/or their expression in the meiotic male germline. In particular, meiotic sex chromosome inactivation (MSCI) has been widely considered a driving force behind depletion of spermatocyte-biased *X*-linked genes in *Drosophila *by analogy with mammals, even though the existence of global MCSI in *Drosophila *has not been proven.

**Results:**

Microarray-based study and qRT-PCR analyses show that the dynamics of gene expression during testis development are very similar between *X*-linked and autosomal genes, with both showing transcriptional activation concomitant with meiosis. However, the genes showing at least ten-fold expression bias toward testis are significantly underrepresented on the *X *chromosome. Intriguingly, the genes with similar expression bias toward tissues other than testis, even those not apparently associated with reproduction, are also strongly underrepresented on the *X*. Bioinformatics analysis shows that while tissue-specific genes often bind silencing-associated factors in embryonic and cultured cells, this trend is less prominent for the *X*-linked genes.

**Conclusions:**

Our data show that the global meiotic inactivation of the *X *chromosome does not occur in *Drosophila*. Paucity of testis-biased genes on the *X *appears not to be linked to reproduction or germline-specific events, but rather reflects a general underrepresentation of tissue-biased genes on this chromosome. Our analyses suggest that the activation/repression switch mechanisms that probably orchestrate the highly-biased expression of tissue-specific genes are generally not efficient on the *X *chromosome. This effect, probably caused by dosage compensation counteracting repression of the *X*-linked genes, may be the cause of the exodus of highly tissue-biased genes to the autosomes.

## Background

Traffic of the newly-created testis-specific genes from the *X *chromosome to autosomes *via *retroposition is a major trend conserved between different taxa [1-3]. In mammals, it is believed to be an adaptation to the evolutionarily 'young' meiotic sex chromosome inactivation (MSCI), so that the newly-duplicated autosomal genes 'rescue' the silencing of the parental *X*-linked genes in the male germline [4]. This process would lead to the loss of the *X*-linked genes that function specifically in the MSCI-affected male germline cells, namely spermatocytes. Indeed, spermatocyte-specific genes are severely underrepresented on the *X *chromosome in mice, illustrating the proposed role of MSCI in genome evolution. In contrast, the male-specific genes expressed at either earlier or later stages of spermatogenesis (that is in spermatogonia or in round spermatids) are over-represented on the mouse *X *chromosome [5,6], consistent with the vigorous selection for male-beneficial *X*-linked genes proposed previously [7].

Reports on the existence of MSCI as a mechanism of global inactivation of the *X*-linked genes in spermatocytes of *Drosophila *are not conclusive. In flies, similar to mammals, spermatocytes rapidly proceed through the S-phase and enter extended prophase I of meiosis, which lasts several days (reviewed in [8]). The premature cytological condensation of the *X *chromosome in primary spermatocytes and the dominant male sterility frequently caused by *X*-autosomal translocations were originally put forward as indicators of MSCI in *Drosophila *[9]. The overall paucity of *X*-linked testis-specific genes and the 'exodus' of the newly-duplicated testis-specific genes from the *X *chromosome *via *retroposition have been reported in flies and interpreted as additional evidence of MSCI [1,10-14]. However, a recent study indicates that retroposing genes avoid the *X *regardless of their expression pattern, and probably owing to their intrinsic integration preferences [15]. The finding that the *X*-linked transgenes driven by a testis-specific *ocn *promoter fail to show active expression [16] further supported the MSCI model, even though the 'sampling' of chromosomes by the transgene insertions was not very comprehensive. Finally, microarray analyses of dissected testes showed lower *X*-linked gene expression in the sample enriched with spermatocytes as compared to samples that included earlier or later developmental stages [17]. However, this finding could have been affected by differences in the somatic cell content between different regions of testis because somatic cells are underrepresented in the spermatocyte-enriched sample; therefore, the genes expressed in somatic cells, but not in germline, could be perceived as inactivated in spermatocytes. On the other hand, no significant dearth of *X*-linked transcription has been detected in another genome-wide analysis of gene expression in gonads, arguing against global inactivation of the *X *chromosome in the meiotic germline [18]. However, active gene expression in spermatogonia and somatic testis cells [19] present in the whole adult testes could in principle 'mask' the putative gene silencing in meiotic cells in this study.

In order to understand better the genome evolution and structure, and to obtain further insight into the tissue-specific gene expression, it is important to determine whether the paucity of male-biased *X*-linked genes in flies is due to MSCI or other novel, yet unknown mechanism(s). To determine whether the expression of *X*-linked genes changes concomitant with meiosis, we performed a study of the testis transcriptome in larval development, tracing the maturation of the first wave of spermatocytes through the third instar and up to the meiotic divisions at the beginning of pupation [20]. In this model, the 'load' of spermatogonia and associated somatic cells is similar between the developmental stages; therefore the differences in gene expression are attributable to an increase in the number of spermatocytes and to their maturation. While the expression of testis-specific *X*-linked genes steadily increased during spermatocyte maturation as expected, global gene expression on the *X *chromosome did not significantly differ from the autosomes throughout the entire period - both results being consistent with the lack of global MSCI. Further, we found that the paucity of testis-biased genes is not a unique example and that other tissue-biased genes are also underrepresented on the *X *chromosome, indicative of this trend not being limited to the reproduction-related genes. Our analyses suggest that the paucity of *X*-linked tissue-specific genes may be driven by the general inefficiency of stringent tissue-specific gene regulation on the *X *chromosome.

## Results and Discussion

### *X*-linked and autosomal testis-biased genes show similar longitudinal profiles of expression in testis development

MSCI should lead to a paucity of the spermatocyte-specific genes on the *X *chromosome, as has been shown in mammals [5]. In *Drosophila*, the vast majority of the testis-specific genes analyzed to date are activated in primary spermatocytes, that is in the meiotic prophase I [21]. To determine whether expression profiles of *X*-linked genes deviate from this trend, we quantitatively analyzed the transcription of eight previously characterized testis-biased *X*-linked genes in larval testis development, using real-time RT-PCR. In parallel, 18 autosomal testis-biased genes were analyzed as a reference group (Table 1) [22-25].

**Table 1:** The *X*-linked and autosomal spermatocyte-specific genes analyzed for their temporal expression during testis development

Gene	Autosomal	X-linked	Reference
*Sdic*		+	[22]
*CG15450*		+	[12]
*CG1314*		+	[12]
*CG1338*		+	[12]
*CG15711*		+	[12]
*CG15452*		+	[12]
*CG11227*		+	[12]
*CG1324*		+	[12]
*Dj*	+		[23]
*fzo*	+		[24]
β(2)Tubulin	+		[25]
*ocn*	+		[16]
*CG6262*	+		[12]
*CG3483*	+		[12]
*CG7813*	+		[12]
*CG15874*	+		[12]
*CG3494*	+		[12]
*CG16837*	+		[12]
*CG4439*	+		[12]
*CG4750*	+		[12]
*CG15873*	+		[12]
*CG15925*	+		[12]

**Figure 1:**
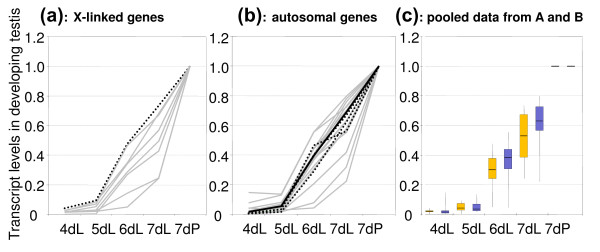
***X*-linked and the autosomal testis-biased genes show similar patterns of activation in testis development**. (A) *X*-linked genes; the profile for the known spermatocyte-specific gene *Sdic *is outlined as a black dotted line. Other genes are *CG15450*, *CG1314*, *CG1338*, *CG15711*, *CG15452*, *CG11227 *and *CG1324 *(gray lines). (B) autosomal genes; the profiles for the known spermatocyte-specific genes *fzo*, *dj*, and β(2)Tubulin are outlined as black dotted lines. The profile for the gene *ocn *which is the source of promoter in the transgene based study of Hense et al. [15] is outlined as a black solid line. Other genes are *CG6262*, *CG3483*, *CG7813*, *CG3492*, *CG15874*, *CG3494*, *CG16837*, *CG4439*, *CG4750*, *CG15873*, *CG15925*, *CG7848*, *CG15710*, *Eyc*, *Mst35Ba*, and *Mst35Bb *(gray lines). (C) Box plot analysis of gene expression data (A, B) shows no significant differences in the expression patterns between the *X*-linked (orange) and the autosomal (blue) gene sets. For each gene, expression level in pupae served as the reference.

Testes were collected daily from day four (second instar) through day seven (Figure 1, dL), at which point third-instar larvae started to pupate (Figure 1, dP). Since the first wave of germline differentiation reaches meiotic divisions approximately at the onset of pupation [19] and spermatocytes become the predominant cell type in testes of third instar larvae [26], this analysis traced changes in gene expression associated with the accumulation and maturation of spermatocytes. Accordingly, transcripts of known spermatocyte-specific genes, both autosomal and *X*-linked, showed a steady increase throughout testis development in our series of samples (dotted lines on Figure 1A, B). Other analyzed genes, including those located on the *X *chromosome, showed the same pattern indicating that their activation occurs in the meiotic germline. The pooled data also show a striking similarity of the expression patterns between the groups of *X*-linked and autosomal genes (Figure 1C). We further sought to determine whether the relatively small selection of *X*-linked genes for our study could have influenced our conclusion that these genes are usually activated concomitant with meiosis. To assess a larger sample, we analyzed published *in situ *hybridization data for 501 *X*-linked genes [21] and found that 474 of them (94%) are induced in primary spermatocytes. Cumulatively, the above data obtained by different approaches convincingly show that numerous *X*-linked genes are activated in the male meiotic germline. It is not clear then why the transgenes driven by promoter of the testis-specific *ocn *gene could not show efficient expression when integrated into the *X *chromosome [16], given that *ocn *shows the same expression profile as endogenous *X*-linked testis-biased genes (compare the solid line on Figure 1B for *ocn *to the profiles in Figure 1A). However, regardless of the mechanism(s) of silencing, the *ocn *transgenes [16] do not appear representative of the *X*-linked genes that are commonly expressed in spermatocytes.

### Global gene expression on the *X *chromosome does not significantly change during testis development

**Figure 2:**
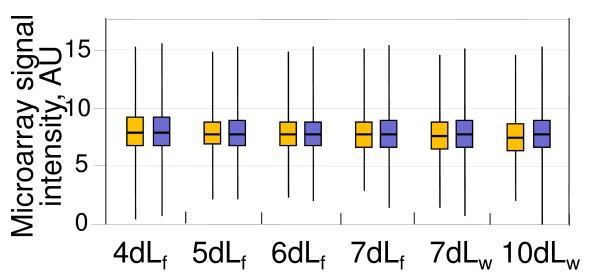
**Lack of global *X*-chromosome inactivation in developing testes**. Expression of *X*-linked (orange) and autosomal (blue) genes was measured as signal intensity of corresponding microarray probes, after normalization. cDNAs hybridized to the microarrays were isolated from testes of either feeding (f) or wandering (w) larvae grown for the indicated number of days at 18°C. The analysis traces the first wave of germline differentiation; pupation indicative of the meiotic divisions occurred at day 11.

To further support our findings by extending them to the majority of *X*-linked genes, we analyzed the global trends of *X*-linked gene expression during testis development using gene microarrays. To improve the resolution of the analysis, we cultured larvae at a lower temperature (18°C) and collected testes from feeding larvae at fourth, fifth, sixth and seventh days of development (Figure 2 dL_f_), and from wandering larvae at days seven and ten, just before the onset of pupation at days 11 to 12 (Figure 2, dL_w_). Throughout larval testis development, we did not find appreciable differences in expression between the *X *chromosome and autosomes (Figure 2, orange *versus *blue bars) or any substantial reduction in *X*-linked gene expression (Figure 2, orange bars), strengthening the hypothesis that the *X *chromosome is not affected by global MSCI in *Drosophila*, contrary to mammalian spermatogenesis.

### Weak testis bias of expression shown by *X*-linked genes

**Figure 3:**
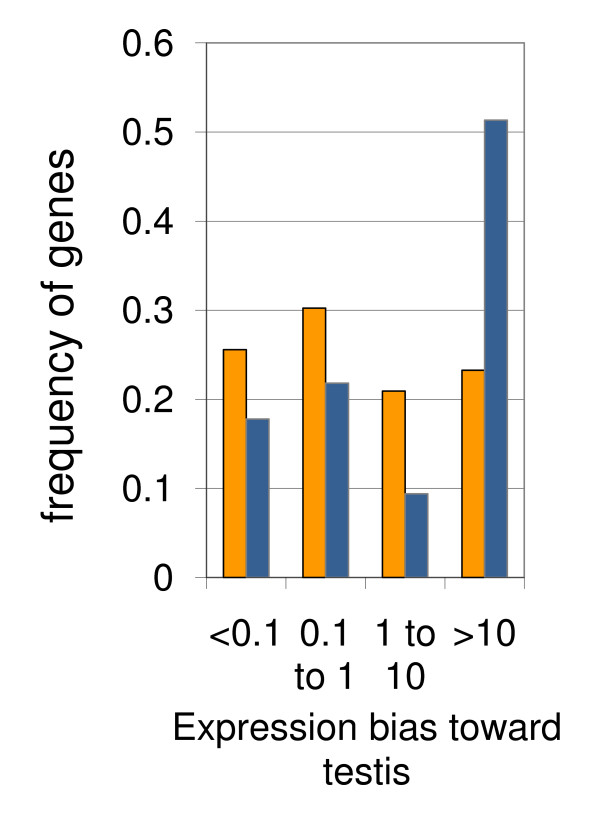
**Paucity of X-linked genes with high expression bias toward testis**. The testis::somatic tissue ratio of microarray signal intensities, observed between adult testis and a panel of somatic tissues [24], serves as a measure of testis bias of gene expression and is shown at bottom. Bars indicate proportion of genes up-regulated in testis development in the four bias categories. Orange bars, *X*-linked genes; blue bars, autosomal genes.

Although we did observe numerous *X*-linked genes increasing their expression during testis development, previous reports showed a paucity of male-biased genes on the *X *[12-14]. To define the relationship between these two gene categories, we analyzed expression bias of the genes up-regulated in developing testes. Using published gene expression data [27], we compared microarray signals observed with adult testis-derived RNA to the signals obtained with RNA from a variety of somatic sources including accessory glands, brain, head, thoracicoabdominal ganglion, crop, midgut, hindgut, malpigian tubules of adults and larvae, ovary, salivary glands, and carcass. Conservatively, the minimal testis-to-somatic tissue signal ratio across the entire panel of analyzed samples was defined as a measure of bias of gene expression toward testis. This analysis identified about 50% of the autosomal genes up-regulated in testis development as highly testis-biased (minimal testis::somatic tissue signal ratio ≥10) (Figure 3, blue bars). Strikingly, highly-biased genes constitute less than one-quarter of the *X*-linked genes, and this difference from the autosomal genes is highly significant (χ^2 ^test, *P *= 3 × 10^-10^) (Figure 3, orange bars). Apparently, it is the cohort of highly testis-biased genes that is depleted in the pool of the *X*-linked genes up-regulated in testis development. As a result, on average the *X*-linked genes show a weaker testis bias of expression than their autosomal counterparts.

### Diverse tissue-specific genes are underrepresented on the *X *chromosome

**Figure 4:**
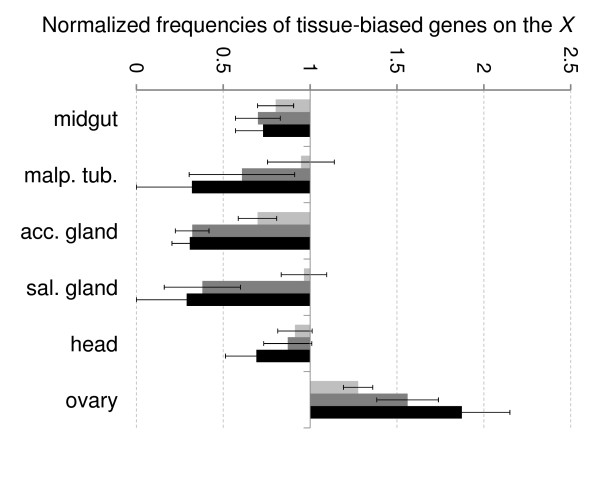
**Highly tissue-biased genes show a skewed representation on the X chromosome**. The ratio of microarray signal intensities observed between the tissue sample indicated at bottom including midgut, malpigian tubule, accessory gland, salivary gland, head, and ovary and a panel of other tissue samples [24] was used as a measure of tissue bias. The bars correspond to the frequencies of the genes on the X chromosome normalized against the genome averages, and are shown for the genes with at least two-fold expression bias toward indicated tissues (light gray), for the genes with at least five-fold bias (dark gray), and for highly biased genes with at least ten-fold bias (black).

Our gene expression study on developing testes indicated that the paucity of testis-biased genes on the *X *chromosome is not caused by the spermatocyte-specific events. We therefore hypothesized that the underlying mechanisms may be not restricted to the male meiotic germline and instead could operate in diverse tissues, causing broad effects on tissue-biased expression. To test this suggestion, using the published gene expression dataset [27] we identified genes that show expression bias toward midgut, malpigian tubules, accessory gland, salivary gland, head, and ovary, using the same bioinformatics approach as described above for testis. We further determined whether the frequencies of the identified genes on the *X *chromosome deviated from the genome averages. We observed that the majority of the analyzed genes show the same trends that the testis-biased genes do: they are underrepresented on the *X *chromosome, and the higher the expression bias the stronger the underrepresentation (Figure 4). One important exception, however, was the ovary-biased gene set for which both trends were reversed. This finding is consistent with previous reports on the overrepresentation of female- and ovary-biased genes on the *X *[13,28], and indicates that the genes selectively expressed in the ovary are subject to unique selective pressures, probably owing to their female-beneficial sexually-antagonistic effects [7,29] and/or because of their peculiar regulation, as discussed below.

### Genes activated in testis development appear to be repressed in embryos and cultured somatic cells

**Figure 5:**
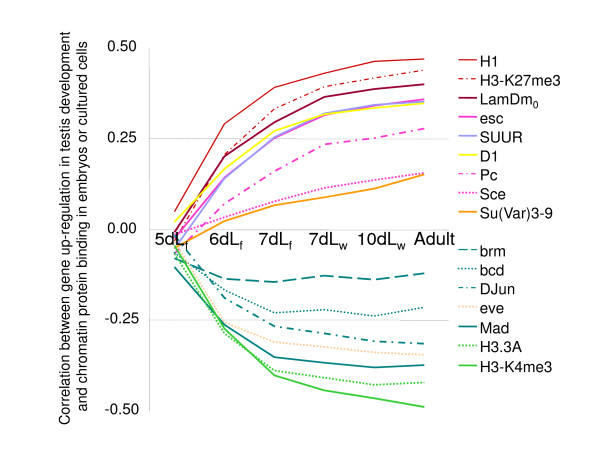
**'Inversed' correlations between gene regulation in testis development and chromatin modifications in somatic cells**. For each of the developmental time points indicated on the horizontal axis, correlations were calculated between up-regulation of genes in testis relative to the earliest time point (from microarray data) and binding of the same genes to the shown chromatin proteins in embryos or somatic cells [29,30]. Yellow, orange, and purple colors represent proteins that are generally associated with gene silencing, and blue and green colors represent proteins generally associated with active gene expression.

Based on our analyses, we suggest that the *X *chromosome provides an inferior environment for specialized genes with expression highly biased toward particular differentiated cell types. To gain a better understanding of the underlying mechanisms, we analyzed the binding of diverse chromatin proteins to the *X*-linked and autosomal tissue-biased genes. First, we analyzed the correlations between gene expression in testis development and binding of 27 chromatin proteins in embryonic and cultured cells. Changes in gene expression were measured as signal fold changes for every time point in our microarray-based analysis; the earliest analyzed stage (four-day old larvae) served as the reference. Chromatin protein binding was assessed as the fold enrichment in chromatin immunoprecipitation or in DNA adenine methyltransferase identification experiments [30,31]. We have found a correlation that was 'inversed' with respect to the major roles of the analyzed proteins: that is, gene up-regulation in testis development was positively correlated with the binding of silencing-associated proteins in embryos and cultured cells (Figure 5, results for proteins with insignificant correlations are not shown). Specifically, testis-up-regulated genes showed prominent associations with histone H1, histone H3 trimethylated at K27, LamDm_o_, D1, and Polycomb (Pc) and Pc group proteins esc and Sce [25,32-38]. In addition, a negative correlation was apparent between genes up-regulated in testis and the binding of gene activation-associated proteins in embryos and cultured cells (these proteins include histone variant H3.3A, H3 trimethylated at K4, DJun, and bcd) [39-41]. These observations imply that testis-biased expression is regulated by a two-prong 'switch' mechanism comprised of gene activation in testis (most likely, male germline) and gene repression in other tissues and cell types.

### Diverse tissue-biased autosomal genes, unlike their *X*-linked counterparts, show strong evidence for repression in embryos and cultured cells

**Figure 6:**
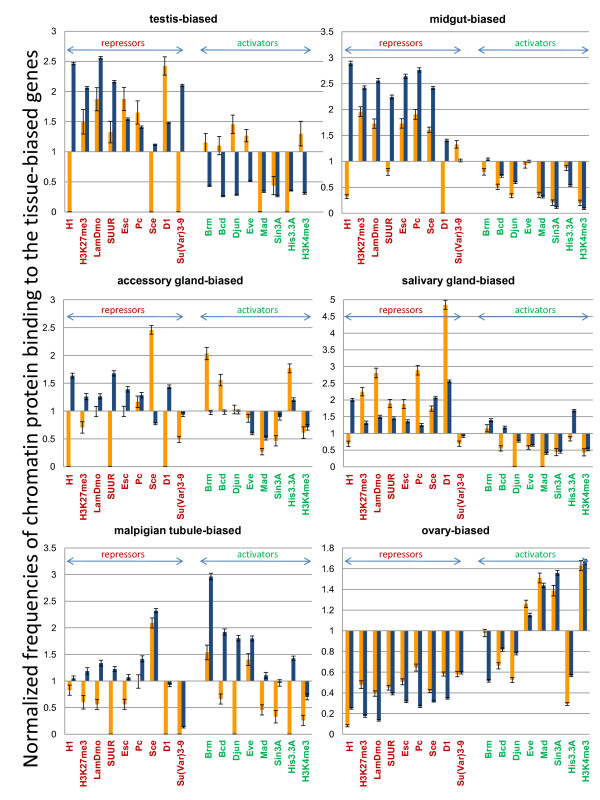
**The autosomal and the *X*-linked tissue-biased genes show different patterns of chromatin modifications**. The frequencies of binding targets for the proteins indicated at the bottom were calculated among the *X*-linked (orange) and autosomal (blue) tissue-biased genes. The sets of genes showing at least two-fold expression bias toward the indicated tissues (such as testis, midgut, accessory gland, salivary gland, malpigian tubule, and ovary) were generated from the genome-wide expression data [24]. Protein binding genes were defined using chromatin immunoprecipitation and DamID data [29,30] with arbitrarily set thresholds. Bars show relative increase or decrease in binding target frequency within analyzed gene sets as compared to the entire genome.

We inquired whether the tissue-biased gene activation generally correlates with binding of repressor proteins in the 'non-target' embryonic and cultured cells, and whether such correlation differs between the *X *chromosome and autosomes. For this analysis, we calculated the relative enrichment with chromatin proteins for the genes that show more than two-fold expression bias toward testis, midgut, accessory gland, salivary gland, malpigian tubule, and ovary. The tissue expression bias was determined using the same approach as described above for the analysis of tissue-biased gene frequencies on chromosomes. Using arbitrarily-set thresholds to define the categories of bound *versus *non-bound genes for each of the analyzed chromatin proteins [30,31], we determined the frequencies of bound genes in the sets of tissue-biased genes on the *X *chromosome and autosomes and normalized them to the frequencies of bound genes in the entire genome. The study included nine repressor proteins and eight activators that showed the strongest 'inverse' correlations in previous analysis. The resulting graphs (Figure 6) show whether the *X*-linked (orange bars) or autosomal (blue bars) genes bind repressors or activators in embryos and cultured cells with a frequency different from the genome average (defined as 1.0).

Consistent with the above correlation studies, this analysis showed that autosomal testis-biased genes are enriched with repressors and rarely bind activators in embryos and cultured cells. In the case of the *X*-linked genes this trend is reduced for the vast majority of the analyzed proteins and even reversed for three repressors and five activators (Figure 6). Therefore, the drastic differences in expression status between germline and somatic cells are less common for the testis-biased genes located on the *X*, indicating that the activation/repression 'switch' is not prominent in the regulation of these genes. Similar observations were made for the majority of genes with an expression bias toward other tissues, with a few exceptions including the malpigian tubule-biased autosomal genes showing enrichment with activators, and the salivary gland-biased *X*-linked genes showing strong enrichment with repressors (Figure 6). In general however, we found that, unlike their autosomal counterparts, the *X*-linked tissue-biased genes are not enriched with repressors and do not lack activators in embryos and cultured cells, implying that the tissue-biased up-regulation of the *X*-linked genes is usually not complemented by their repression in other, 'non-target' cell types. In this respect, ovary-biased genes may provide an example of tissue-biased genes that apparently do not rely on the activation/repression mechanism, regardless of their chromosomal location, because, unlike other tissue-biased genes, they are strongly associated with activators and do not bind repressors in embryonic and cultured cells. This unique feature may facilitate accumulation of the ovary-biased genes on the *X *chromosome.

## Conclusions

Our findings presented in this study strongly suggest that global inactivation of the *X *chromosome does not occur in the *Drosophila *meiotic male germline, and therefore other factors and mechanisms should be invoked to explain the peculiarities of *X*-linked gene expression. A study of gene expression in testis development using microarrays and qRT-PCR, combined with analysis of published *in situ *hybridization data convincingly show that the majority of *X*-linked genes do not undergo silencing concomitant with meiosis; in fact, many of them are activated in the meiotic germline cells. In agreement with the analysis of sex-biased expression in several *Drosophila *species [18], we conclude that the possible appearance of *X*-chromosome underexpression in male gonads results from the paucity of *X*-linked testis-biased genes rather than from chromosome-wide gene silencing. Why the *X *chromosome behaves in meiotic cells in *Drosophila *unlike in mammals is not clear; perhaps the yet unidentified mechanisms that cause overexpression of *X*-linked genes in the *Drosophila *germline [18] counteract global silencing of the *X *chromosome.

Our findings could be consistent with the sexual antagonism driving *X *inactivation (SAXI) model [10], in which the pressure of sexual selection drives the 'exodus' of male germline-specific genes from the *X *chromosome causing its inactivation in meiotic cells, with the one exception that no true global *X *inactivation is seen, just the lack of testis-biased genes on the *X*. However, a recent study indicates that retroposing genes with diverse expression biases all show an affinity toward autosomes [15]; therefore the apparent 'exodus' of male-biased genes from the *X*, previously considered as the major indirect evidence for MSCI in flies [1], is not necessarily driven by sexual selection. Earlier studies did show that not only testis-biased, but also somatic male-biased genes are underrepresented on the *X *[42], but the possible role of these genes in sexual selection could still be invoked with certain ease owing to their sex bias. Here, we showed that the genes with strong expression bias toward tissues that are not directly related to reproduction, such as salivary glands, midgut, and malpigian tubules, also show paucity on the *X *chromosome. Taking into account the recent report that somatic gene expression may be regulated by gonads [43], we inquired whether the gonad-dependent genes appreciably contributed to our tissue-biased gene sets. The heaviest representation of gonad-dependent genes [43] was found, not surprisingly, in the testis-biased and ovary-biased gene sets but in both cases it was less than 4%, and less than 2% of the genes preferentially expressed in salivary glands, midgut, or malpigian tubules appear to be gonad-dependent. Thus, the paucity of tissue-biased genes, and probably the underlying 'exodus' of such genes from the *X *chromosome, involves genes that have no apparent specific role in reproduction.

A possible clue to the reasons why tissue-biased genes are so scarce on the *X *is provided by the finding that the genes with the highest bias are affected much more than the genes with modest expression differences (Figure 4B). Based on the analysis of chromatin protein binding to the biased genes, we hypothesize that the high expression bias results from the combined effect of gene activation in the 'target' cells and repression in other cell types. Our analysis indicates that such a mechanism does not operate frequently on the *X*-linked tissue-biased genes. This finding presents 'the chicken or the egg' dilemma because we cannot discern whether the weak tissue bias of expression results from not using the activation/repression 'switch' mechanism, or *vice versa*. Nevertheless, it is possible that the inefficiency of this 'switch' on the *X *chromosome significantly affects genome evolution. The importance of this influence is illustrated by the unique example of the ovary-biased genes that apparently do not rely on the activation/repression mechanism (Figure 6) and strongly accumulate on the *X *chromosome (Figure 4) in perfect agreement with the female-beneficial selection model [7,29].

Of the two components of the activation/repression switch, the repression may be vulnerable to the *X *chromosome-specific mechanism of dosage compensation that broadly and ubiquitously activates *X*-linked genes in somatic cells [44]. To test this suggestion, we analyzed binding of the testis-up-regulated genes with the key dosage compensation component msl-2 using published datasets [45,46] expecting to find that the highly testis-biased genes do not bind msl-2. We found no correlation between testis bias of expression and msl-2 binding. However, dosage compensation machinery can regulate genes at a distance from the primary binding sites [47] and a recent study did show that the genes with the highest male bias of expression tend to localize outside the compensated regions [48]. We propose, therefore, that the H4-K16 acetylation of the *X *chromosome by the dosage compensation complex interferes with highly tissue-biased gene expression by counteracting repression in the 'non-target' cell types. This may present a significant problem if the gene product is toxic to diverse cells (which for example could be expected for many sperm differentiation genes), and could result in a strong selective pressure toward relocation of the highly tissue-biased genes to the autosomes.

## Methods

### Fly culture and dissection

Wild type (Oregon) larvae were grown on yeast-molasses media at 18°C. At the specified time points larvae were collected, and testes harvested using manual dissection. Further analyses utilized samples pooled from at least 30 larvae.

### Quantitative RT-PCR

Total RNA was isolated from dissected testes with Trizol reagent (Invitrogen, Carlsbad, CA, USA), and cDNA synthesized from 1 μg RNA samples using oligo(dT) primer and SuperScript II reverse transcriptase (Invitrogen). Real-time PCR was performed in the iCycler iQ5 instrument (Bio-Rad, Hercules, CA, USA) using SYBR Green chemistry; *rp49 *transcripts served as template loading reference.

### Microarray probes

Total RNA was isolated from testes with Trizol reagent (Invitrogen), and further purified with the RNeasy kit (Qiagen, Hilden, North Rhine-Westphalia, Germany). Poly(A)+ RNA was selectively amplified from 0.5 to 1 μg total RNA samples with the BD SMART cDNA Synthesis kit and the BD Advantage 2 PCR Enzyme System (Clontech, Mountain View, CA, USA). Amplified cDNAs were cleaned with the Wizard SV Gel kit (Promega, Madison, WI, USA). cDNA samples of 3.5 to 4.5 μg were labeled with the ULYSIS Alexa Fluor 546 or Alexa Fluor 647 (Molecular Probes, Eugene, OR, USA) dyes according to the manufacturer's protocol, and used for competitive hybridization with microarrays.

### Hybridization of samples to microarrays

The *Drosophila *oligonucleotide microarray set (Qiagen-Operon) was printed on aminosilane-coated slides at Tufts-New England Medical Center Expression Array Core facility (TEAC). The set contains 14,593 70-mer oligonucleotides representing 13,664 genes which cover most of genes in release 3.2 of the *Drosophila *genome (The FlyBase Consortium, 2003). The probe sequences from the Operon set were re-mapped to *Drosophila *genome release 5.1 using BLAST, and probes with multiple hits or ambiguous mapping were excluded from subsequent analysis. Additional information on the *Drosophila *oligonucleotide set can be found at the manufacturer's website http://www.operon.com. Details of the array design can be found at ArrayExpress (array design Operon AROS D. melanogaster v.1.1). Labeled pairs of samples were hybridized in 1 × hybridization buffer (GE Healthcare, Little Chalfont, Buckinghamshire, UK), 20% formamide, and 0.025% each of Ficoll, polyvinylpyrrolidone, Na pyrophosphate, and heparin. Samples were incubated with microarrays for 44 hours at 37°C. After hybridization slides were washed, dried by centrifugation, and scanned on the ScanArray 4000 scanner (PerkinElmer, Waltham, MA, USA).

### Data acquisition and analysis

Fluorescence intensities of individual spots were acquired from the array images with ImaGene software (BioDiscovery). Subsequent normalization and statistical analysis were performed with package *limma *[49,50], part of the BioConductor project [51]. Normalization of the data is described in Normalization protocol associated with ArrayExpress submission E-MEXP-1980 of the microarray experiment described in this manuscript. At least three hybridization replicates passing quality control were performed per experimental condition. Data were analyzed using the linear modeling approach, and Bayes moderated t test was used to determine differential expression between each time point and the fourth day (the earliest time point). q values for individual contrasts represent FDR for multiple testing [52]. q < 0.1 was chosen as the threshold for differential expression unless specified differently.

Hypergeometric analysis of the representation of differentially expressed genes on *Drosophila *chromosomes was performed with the web-based tool GeneMerge (http://genemerge.cbcb.umd.edu/[53]). Significance of the differences in bin content for different chromosome arms (see Figure 4 and related text in Results section) was assessed with Fisher's exact test as implemented in R, and correlations between the gene expression and protein binding data were calculated with two-sided Pearson's method, also as implemented in R [54]. The significance of Fisher's and Pearson's tests was adjusted for multiple testing according to Bonferroni method within corresponding data series.

## Abbreviations

MSCI: meiotic sex chromosome inactivation; qRT-PCR: reverse transcription followed with quantitative polymerase chain reaction analysis; FDR: false discovery rate.

## Authors' contributions

DIN designed research; LMM performed research; LMM and DIN analyzed data; and DIN wrote the paper. All authors read and approved the final manuscript.

## References

[B1] Betrán E Thornton K Long M Retroposed new genes out of the *X *in *Drosophila* Genome Res 2002 12 1854 1859 10.1101/gr.6049 12466289 PMC187566

[B2] Emerson JJ Kaessmann H Betrán E Long M Extensive gene traffic on the mammalian *X *chromosome Science 2004 303 537 540 10.1126/science.1090042 14739461

[B3] Vinckenbosch N Dupanloup I Kaessmann H Evolutionary fate of retroposed gene copies in the human genome Proc Natl Acad Sci USA 2006 103 3220 3225 10.1073/pnas.0511307103 16492757 PMC1413932

[B4] Potrzebowski L Vinckenbosch N Marques AC Chalmel F Jégou B Kaessmann H Chromosomal gene movements reflect the recent origin and biology of therian sex chromosomes PLoS Biol 2008 6 e80 10.1371/journal.pbio.0060080 18384235 PMC2276528

[B5] Khil PP Smirnova NA Romanienko PJ Camerini-Otero RD The mouse *X *chromosome is enriched for sex-biased genes not subject to selection by meiotic sex chromosome inactivation Nat Genet 2004 36 642 646 10.1038/ng1368 15156144

[B6] Mueller JL Mahadevaiah SK Park PJ Warburton PE Page DC Turner JM The mouse *X *chromosome is enriched for multicopy testis genes showing postmeiotic expression Nat Genet 2008 40 794 799 10.1038/ng.126 18454149 PMC2740655

[B7] Rice WR Sex chromosomes and the evolution of sexual dimorphism Evolution 1984 38 735 742 10.2307/2408385 28555827

[B8] White-Cooper H Molecular mechanisms of gene regulation during Drosophila spermatogenesis Reproduction 2010 139 11 21 10.1530/REP-09-0083 19755484

[B9] Lifschytz E Lindsley DL The role of *X*-chromosome inactivation during spermatogenesis Proc Nat Acad Sci USA 1972 69 182 186 10.1073/pnas.69.1.182 4621547 PMC427571

[B10] Wu C-I Xu EY Sexual antagonism and *X *inactivation--the SAXI hypothesis Trends Genet 2003 19 243 247 10.1016/S0168-9525(03)00058-1 12711214

[B11] Gurbich TA Bachtrog D Gene content evolution on the *X *chromosome Curr Opin Genet Dev 2008 18 493 498 10.1016/j.gde.2008.09.006 18929654 PMC4590997

[B12] Boutanaev AM Kalmykova AI Shevelyov YY Nurminsky DI Large clusters of co-expressed genes in the *Drosophila *genome Nature 2002 420 666 669 10.1038/nature01216 12478293

[B13] Parisi M Nuttall R Naiman D Bouffard G Malley J Andrews J Eastman S Oliver B Paucity of genes on the *Drosophila X *chromosome showing male-biased expression Science 2003 299 697 700 10.1126/science.1079190 12511656 PMC1363366

[B14] Sturgill D Zhang Y Parisi M Oliver B Demasculinization of *X *chromosomes in the *Drosophila *genus Nature 2007 450 238 241 10.1038/nature06330 17994090 PMC2386140

[B15] Metta M Schlötterer C Non-random genomic integration - an intrinsic property of retrogenes in Drosophila? BMC Evol Biol 2010 10 114 10.1186/1471-2148-10-114 20426838 PMC2879276

[B16] Hense W Baines JF Parsch J *X *chromosome inactivation during *Drosophila *spermatogenesis PLoS Biol 2007 5 e273 10.1371/journal.pbio.0050273 17927450 PMC2001211

[B17] Vibranovski MD Lopes HF Karr TL Long M Stage-specific expression profiling of *Drosophila *spermatogenesis suggests that meiotic sex chromosome inactivation drives genomic relocation of testis-expressed genes PLoS Genet 2009 5 e1000731 10.1371/journal.pgen.1000731 19936020 PMC2770318

[B18] Gupta V Parisi M Sturgill D Nuttall R Doctolero M Dudko OK Malley JD Eastman PS Oliver B Global analysis of *X*-chromosome dosage compensation J Biol 2006 5 3 10.1186/jbiol30 16507155 PMC1414069

[B19] Olivieri G Olivieri A Autoradiographic study of nucleic acid synthesis during spermatogenesis in *Drosophila melanogaster* Mutat Res 1965 2 366 380 5878312 10.1016/0027-5107(65)90072-2

[B20] Lindsey DL Tokuyasu KT Ashbumer M, Wright TRF Spermatogenesis The Genetics and Biology of Drosophila 1980 2 London: Academic Press 226 294

[B21] Zhao J Klyne G Benson E Gudmannsdottir E White-Cooper H Shotton D FlyTED: the *Drosophila *Testis Gene Expression Database Nucl Acids Res 2010 38 D710 D715 10.1093/nar/gkp1006 19934263 PMC2808924

[B22] Nurminsky DI Nurminskaya MV De Aguiar D Hartl DL Selective sweep of a newly evolved sperm-specific gene in *Drosophila* Nature 1998 396 572 575 10.1038/25126 9859991

[B23] Blümer N Schreiter K Hempel L Santel A Hollmann M Schäfer MA Renkawitz-Pohl R A new translational repression element and unusual transcriptional control regulate expression of *don juan *during *Drosophila *spermatogenesis Mech Dev 2002 110 97 112 10.1016/S0925-4773(01)00577-9 11744372

[B24] Hwa JJ Hiller MA Fuller MT Santel A Differential expression of the *Drosophila *mitofusin genes *fuzzy onions *(*fzo*) and *dmfn* Mech Dev 2002 116 213 216 10.1016/S0925-4773(02)00141-7 12128227

[B25] Michiels F Gasch A Kaltschmidt B Renkawitz-Pohl R A 14 bp promoter element directs the testis specificity of the *Drosophila beta 2 tubulin *gene EMBO J 1989 8 1559 1565 2504583 10.1002/j.1460-2075.1989.tb03540.x PMC400987

[B26] Shevelyov YY Lavrov SA Mikhaylova LM Nurminsky ID Kulathinal RJ Egorova KS Rozovsky YM Nurminsky DI The B-type lamin is required for somatic repression of testis-specific gene clusters Proc Natl Acad Sci USA 2009 106 3282 3287 10.1073/pnas.0811933106 19218438 PMC2651240

[B27] Chintapalli VR Wang J Dow JA Using FlyAtlas to identify better *Drosophila melanogaster *models of human disease Nat Genet 2007 39 715 720 10.1038/ng2049 17534367

[B28] Ranz JM Castillo-Davis CI Meiklejohn CD Hartl DL Sex-dependent gene expression and evolution of the *Drosophila *transcriptome Science 2003 300 1742 1745 10.1126/science.1085881 12805547

[B29] Vicoso B Charlesworth B Evolution on the X chromosome: unusual patterns and processes Nat Rev Genet 2006 7 645 653 10.1038/nrg1914 16847464

[B30] Schwartz YB Kahn TG Nix DA Li XY Bourgon R Biggin M Pirrotta V Genome-wide analysis of Polycomb targets in *Drosophila melanogaster* Nat Genet 2006 38 700 705 10.1038/ng1817 16732288

[B31] de Wit E Braunschweig U Greil F Bussemaker HJ van Steensel B Global chromatin domain organization of the *Drosophila *genome PLoS Genet 2008 24 e1000045 10.1371/journal.pgen.1000045 PMC2274884 18369463

[B32] Ni JQ Liu LP Hess D Rietdorf J Sun FL *Drosophila *ribosomal proteins are associated with linker histone H1 and suppress gene transcription Genes Dev 2006 20 1959 1973 10.1101/gad.390106 16816001 PMC1522087

[B33] Kim K Choi J Heo K Kim H Levens D Kohno K Johnson EM Brock HW An W Isolation and characterization of a novel H1.2 complex that acts as a repressor of p53-mediated transcription J Biol Chem 2008 283 9113 9126 10.1074/jbc.M708205200 18258596 PMC2431041

[B34] Schones DE Zhao K Genome-wide approaches to studying chromatin modifications Nat Rev Genet 2008 9 179 191 10.1038/nrg2270 18250624 PMC10882563

[B35] Simon JA Tamkun JW Programming off and on states in chromatin: mechanisms of Polycomb and trithorax group complexes Curr Opin Genet Dev 2002 12 210 218 10.1016/S0959-437X(02)00288-5 11893495

[B36] Czermin B Melfi R McCabe D Seitz V Imhof A Pirrotta V *Drosophila *enhancer of Zeste/ESC complexes have a histone H3 methyltransferase activity that marks chromosomal Polycomb sites Cell 2002 111 185 196 10.1016/S0092-8674(02)00975-3 12408863

[B37] Fritsch C Beuchle D Muller J Molecular and genetic analysis of the Polycomb group gene Sex combs extra/Ring in *Drosophila* Mech Dev 2003 120 949 954 10.1016/S0925-4773(03)00083-2 12963114

[B38] Aulner N Monod C Mandicourt G Jullien D Cuvier O Sall A Janssen S Laemmli UK Kas E The AT-hook protein D1 is essential for *Drosophila melanogaster *development and is implicated in position-effect variegation Molec Cell Biol 2002 22 1218 1232 10.1128/MCB.22.4.1218-1232.2002 11809812 PMC134649

[B39] Ma J Crossing the line between activation and repression Trends Genet 2005 21 54 59 10.1016/j.tig.2004.11.004 15680515

[B40] Mito Y Henikoff JG Henikoff S Genome-scale profiling of histone H3.3 replacement patterns Nat Genet 2005 37 1090 1097 10.1038/ng1637 16155569

[B41] Kockel L Homsy JG Bohmann D *Drosophila *AP-1: lessons from an invertebrate Oncogene 2001 20 2347 2364 10.1038/sj.onc.1204300 11402332

[B42] Sturgill D Zhang Y Parisi M Oliver B Demasculinization of X chromosomes in the *Drosophila *genus Nature 2007 450 238 241 10.1038/nature06330 17994090 PMC2386140

[B43] Parisi MJ Gupta V Sturgill D Warren JT Jallon JM Malone JH Zhang Y Gilbert LI Oliver B Germline-dependent gene expression in distant non-gonadal somatic tissues of *Drosophila* BMC Genomics 2010 11 346 10.1186/1471-2164-11-346 20515475 PMC2887422

[B44] Akhtar A Becker P Activation of transcription through histone H4 acetylation by MOF, an acetyltransferase essential for dosage compensation in *Drosophila* Mol Cell 2000 5 367 375 10.1016/S1097-2765(00)80431-1 10882077

[B45] Alekseyenko AA Larschan E Lai WR Park PJ Kuroda MI High-resolution ChIP-chip analysis reveals that the *Drosophila *MSL complex selectively identifies active genes on the male *X *chromosome Genes Dev 2006 20 848 857 10.1101/gad.1400206 16547173 PMC1472287

[B46] Gilfillan GD Straub T de Wit E Greil F Lamm R van Steensel B Becker PB Chromosome-wide gene-specific targeting of the *Drosophila *dosage compensation complex Genes Dev 2006 20 858 870 10.1101/gad.1399406 16547172 PMC1475731

[B47] Larschan E Alekseyenko AA Gortchakov AA Peng S Li B Yang P Workman JL Park PJ Kuroda MI MSL complex is attracted to genes marked by H3K36 trimethylation using a sequence-independent mechanism Mol Cell 2007 28 121 133 10.1016/j.molcel.2007.08.011 17936709

[B48] Bachtrog D Toda NR Lockton S Dosage compensation and demasculinization of X chromosomes in *Drosophila* Curr Biol 2010 20 1476 1481 10.1016/j.cub.2010.06.076 20705467 PMC4511158

[B49] Smyth GK Gentleman R, Carey V, Dudoit S, Irizarry R, Huber W Limma: linear models for microarray data Bioinformatics and Computational Biology Solutions using R and Bioconductor 2005 New York: Springer 397 420

[B50] Smyth G K Yang Y-H Speed TP Statistical issues in microarray data analysis Methods Mol Biol 2003 224 111 136 12710670 10.1385/1-59259-364-X:111

[B51] Gentleman RC Carey VJ Bates DM Bolstad B Dettling M Dudoit S Ellis B Gautier L Ge Y Gentry J Hornik K Hothorn T Huber W Iacus S Irizarry R Leisch F Li C Maechler M Rossini AJ Sawitzki G Smith C Smyth G Tierney L Yang JY Zhang J Bioconductor: open software development for computational biology and bioinformatics Genome Biol 2004 5 R80 10.1186/gb-2004-5-10-r80 15461798 PMC545600

[B52] Benjamini Y Hochberg Y Controlling the false discovery rate: a practical and powerful approach to multiple testing J R Statist Soc B 1995 57 289 300

[B53] Castillo-Davis CI Hartl DL GeneMerge: post-genomic analysis, data mining, and hypothesis testing Bioinformatics 2003 19 891 892 10.1093/bioinformatics/btg114 12724301

[B54] R Development Core Team R: A Language and Environment for Statistical Computing Vienna: R Foundation for Statistical Computing 2006 http://www.R-project.org

